# Improvement of Hydrogen Desorption Characteristics of MgH_2_ With Core-shell Ni@C Composites

**DOI:** 10.3390/molecules23123113

**Published:** 2018-11-28

**Authors:** Cuihua An, Qibo Deng

**Affiliations:** Tianjin Key Laboratory of Advanced Functional Porous Materials, Institute for New Energy Materials & Low-Carbon Technologies, School of Materials Science and Engineering, Tianjin University of Technology, NO.391 Binshui West Street Xiqing District, Tianjin 300384, China; ancuihua@tjut.edu.cn

**Keywords:** magnesium hydride, dehydrogenation kinetics, Ni@C core-shell nanostructure, hydrogen storage materials, catalytic effect

## Abstract

Magnesium hydride (MgH_2_) has become popular to study in hydrogen storage materials research due to its high theoretical capacity and low cost. However, the high hydrogen desorption temperature and enthalpy as well as the depressed kinetics, have severely blocked its actual utilizations. Hence, our work introduced Ni@C materials with a core-shell structure to synthesize MgH_2_-*x* wt.% Ni@C composites for improving the hydrogen desorption characteristics. The influences of the Ni@C addition on the hydrogen desorption performances and micro-structure of MgH_2_ have been well investigated. The addition of Ni@C can effectively improve the dehydrogenation kinetics. It is interesting found that: i) the hydrogen desorption kinetics of MgH_2_ were enhanced with the increased Ni@C additive amount; and ii) the dehydrogenation amount decreased with a rather larger Ni@C additive amount. The additive amount of 4 wt.% Ni@C has been chosen in this study for a balance of kinetics and amount. The MgH_2_-4 wt.% Ni@C composites release 5.9 wt.% of hydrogen in 5 min and 6.6 wt.% of hydrogen in 20 min. It reflects that the enhanced hydrogen desorption is much faster than the pure MgH_2_ materials (0.3 wt.% hydrogen in 20 min). More significantly, the activation energy (*E*_A_) of the MgH_2_-4 wt.% Ni@C composites is 112 kJ mol^−1^, implying excellent dehydrogenation kinetics.

## 1. Introduction

With the approximate exhaustion of traditional fossil fuel and increasing environment concerns, seeking clean renewable energy has become one of the top priorities for scientific researchers [1,2]. Hydrogen energy is deemed to be a promising candidate to supersede conventional energy due to its non-polluting and reproducible features [3,4,5,6]. After Bogdanović and Schwichardi’s breakthrough, solid-state hydrogen storage materials, especially magnesium hydride (MgH_2_), have become popular to study because of their excellent reversibility, high theoretical capacity (7.6 wt.%) and low cost [7,8,9,10,11,12,13]. Nevertheless, the presence of some obstacles such as high decomposition enthalpy and dehydrogenation temperature, and sluggish kinetics, have definitely hindered further development on the actual utilizations.

Until now, numerous tactics have been put forward and pullulated, aiming at ameliorating the hydrogen storage performances of MgH_2_, including nanocrystallization, alloying and adding catalysts [14,15,16,17,18,19]. In fact, plentiful literatures have shown that introducing suitable catalysts is one of the most effective strategies for decreasing the dehydrogenation temperature and enhancing dehydrogenation kinetics [20,21,22]. Thus, many kinds of catalysts have been characterized for the dehydrogenation of MgH_2_, including transition metals (Ti, V, Fe, Co and Ni, etc.) and their composites [23,24,25,26]. Reports have shown that Ni-based complexes have displayed effective catalytic activity for hydrogen escape from MgH_2_. The composite of MgH_2_ + 5 wt.% Ni/TiO2 is reported to desorb 5.24 wt.% H_2_ in 30 min at 250 °C temperature [27]. Recently, Zhang et al. systematically studied the influence of Ni morphology (including shape and size) on the hydrogen storage performances of MgH_2_, and provided a guideline for designing nanostructured catalysts with high activity [28]. Moreover, the novel carbon structure is more favorable for further improving its catalytic activity of Ni/C compounds [29,30,31,32]. The amount of released hydrogen from the MgH_2_@1Ni-CMK-3 was pointed out by Jia et al. to be as high as 5.8 wt.% within 60 min at 300 °C [33].This could be attributed to the porous nanostructures which provide more transfer channels for the desorption of hydrogen from the bulk of the MgH_2_ materials. Hence, in the present work, the prepared one-dimensional Ni@C nanorods are served as additive and the influences of the additive amount on the dehydrogenation performances of MgH_2_-Ni@C composites are investigated comprehensively.

## 2. Results and Discussion

**Figure 1:**
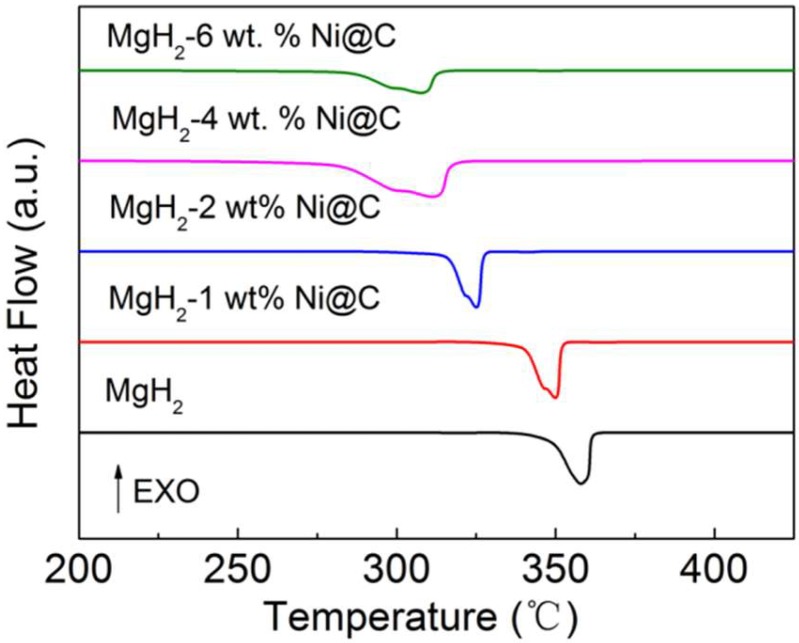
Differential scanning calorimetry (DSC) plots of various MgH_2_-*x* wt.% Ni@C composites (*x* = 0, 1, 2, 4 and 6).

Differential scanning calorimetry (DSC) measurements were conducted to discuss the thermal decomposition properties of MgH_2_-*x* wt.% Ni@C composites (*x* = 0, 1, 2, 4 and 6) and the corresponding DSC curves in the temperature range from 200 °C to 450 °C (5 °C min^−1^ heating rate) are shown in Figure 1. It is evident that both the onset dehydrogenation temperature and dehydrogenation peak temperature gradually decrease with the increased Ni@C additive amount. Table 1 presents the exact values of the onset and peak temperatures which are shown in Figure 1. The exact values of the onset temperature in Table 1 are chosen at the intersection between the DSC plot and the baseline.

The broad dehydrogenation peaks in Figure 1 of the thermal decomposition process could be attributed to the nonuniformity of the particle sizes in MgH_2_. One interesting finding is that the dehydrogenation temperature of the MgH_2_-Ni@C composites is lower than that of pure MgH_2_ materials. This phenomenon indicates that the addition of Ni@C materials can enhance the hydrogenation dynamic properties of MgH_2_.

**Figure 2:**
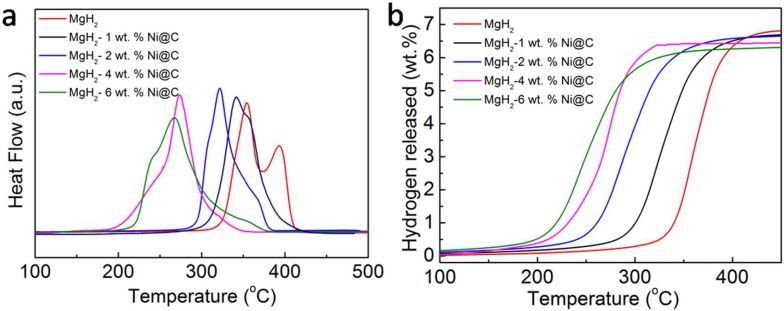
The temperature-programmed desorption (TPD) plots (**a**) and the corresponding thermally programmed H2 desorption capacity curves (**b**) of various MgH_2_-*x* wt.% Ni@C composites (*x* = 0, 1, 2, 4 and 6).

Temperature-programmed desorption system (TPD) tests were then carried out to further investigate the influence of Ni@C additives on the hydrogen desorption performances of MgH_2_. The TPD plots of various additive amounts of MgH_2_-Ni@C composites are reported in Figure 2a. Obviously, there are two hydrogen desorption peaks without Ni@C in the pyrolysis procedure, which is induced by uneven particle distribution. Moreover, the onset and peak temperatures of MgH_2_-Ni@C composite accordingly reduce with increasing Ni@C additive dosage, which is consistent with the DSC results. The onset temperatures of the 4 wt.% and 6 wt.% Ni@C additive dosage reduce to 182 and 191 °C, respectively, which is much lower than that of pure MgH_2_ (302 °C). The peak temperatures with Ni@C additives correspondingly decrease. The dehydrogenation capacities of the pure MgH_2_ and various MgH_2_-*x* wt.% Ni@C (*x* = 1, 2, 4 and 6) composites are 6.8%, 6.7%, 6.6%, 6.4% and 6.3%, respectively. Although the addition of Ni@C materials has distinctly decreased the dehydrogenation temperature and enhanced the hydrogen desorption performances, the amount of hydrogen desorption capacity for MgH_2_-Ni@C composites decreased due to its hydrogen nonabsorbent activities. By comparison, it was found that the dehydrogenation temperatures of composites with 4 wt.% and 6 wt.% Ni@C additive amounts are approximately the same, while the 6 wt.% Ni@C additive amounts exhibited a lower hydrogen desorption capacity. Therefore, MgH_2_-4 wt.% Ni@C composites have been chosen to further survey the micro-structural variation and hydrogen desorption properties. 

**Figure 3:**
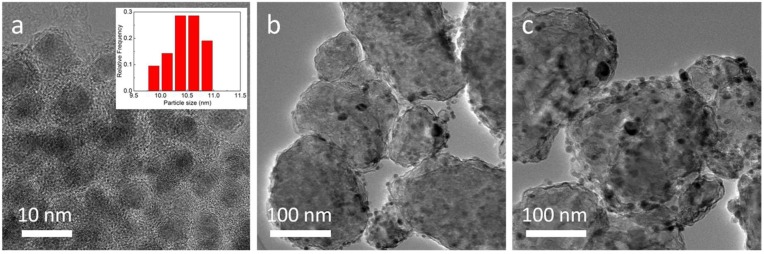
Transmission electron microscopy (TEM) images of Ni@C (**a**) (inset of size distribution), MgH_2_-4 wt.% Ni@C after dehydrogenation (**b**), MgH_2_-4 wt.% Ni@C after five adsorbed-desorbed cycles (**c**).

The morphology and micro-structural features of MgH_2_-4 wt.% Ni@C composites before and after dehydrogenation were characterized by transmission electron microscopy (TEM) analysis (Figure 3). Initially, the Ni@C materials depicted a core-shell microstructure with approximately a 10 nm Ni core and 5 nm carbon shell (Figure 3a). The size distribution (inset of Figure 3a) reflects a relatively uniform distribution. The carbon shell possesses many porous channels which are beneficial to diffusing hydrogen in the composites. In Figure 3b, the MgH_2_-4 wt.% Ni@C composites are assembled by irregular nanoparticles, up to a hundred nm in diameter with numerous Ni@C nanoparticles on it. After five working cycles, the MgH_2_-4 wt.% Ni@C composites maintain the same irregular morphology but the particle size becomes distinctly large (Figure 3c).

This is because the MgH_2_-4 wt.% Ni@C composites have passed through the dissociation, diffusion, nucleation, growth and re-dissociation process of the hydrogen during the cycle. There is interface migration, decomposition and combination of various phases in the above processes. Lastly, these decomposition and re-combination reaction to generate magnesium hydride result in an increase of particle size. 

**Figure 4:**
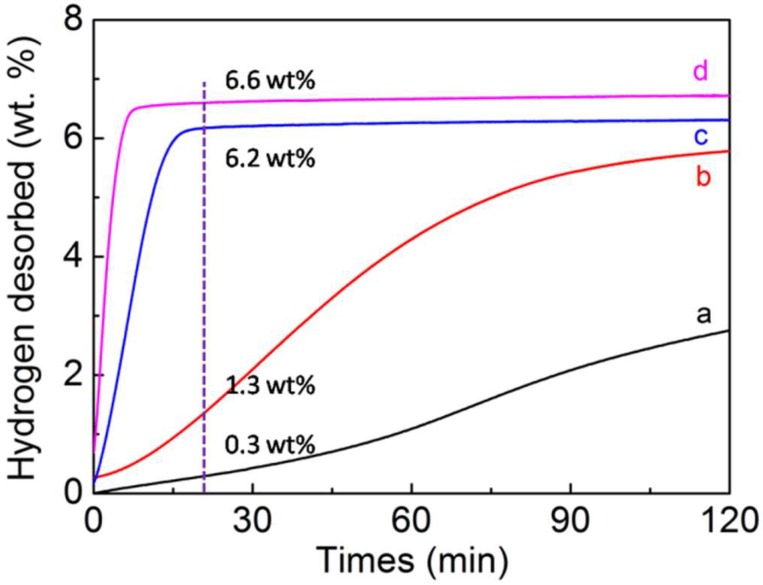
Thermal dehydrogenation performances of pure MgH_2_ at 300 °C (a), MgH_2_-4 wt.% Ni@C composites at 230 °C (b), MgH_2_-4 wt.% Ni@C composites at 270 °C (c), MgH_2_-4 wt.% Ni@C composites at 300 °C (d).

To better understand the hydrogen desorption kinetics of pure MgH_2_ materials and MgH_2_-4 wt.% Ni@C composites, we now discuss the isothermal dehydrogenation curves at 300 °C (Figure 4). Compared to pure MgH_2_, the hydrogen desorption kinetics of MgH_2_-4 wt.% Ni@C composites were raised at the same temperature (300 °C). The MgH_2_-4 wt.% Ni@C composites can release 5.9 wt.% hydrogen in 5 min and 6.6 wt.% hydrogen in 20 min whereas the pure MgH_2_ can only release 0.3 wt.% hydrogen in 20 min and 2.7 wt.% hydrogen in an even longer time of 120 min. Thus, the addition of Ni@C has indeed enhanced the hydrogen desorption kinetic. Meanwhile, the hydrogen desorption kinetics of MgH_2_-4 wt.% Ni@C composites at different temperatures are evaluated in Figure 4. The dehydrogenation kinetics of MgH_2_-4 wt.% Ni@C composites appear to have weakened with the temperature decrease. More specifically, the MgH_2_-4 wt.% Ni@C composites can release 5.8 wt.% of hydrogen in 120 min at 230 °C while 5.98 wt.% of hydrogen is released in 15 min at 270 °C. All experimental data verify that the Ni@C materials exhibit catalytic properties for magnesium hydride. 

**Figure 5:**
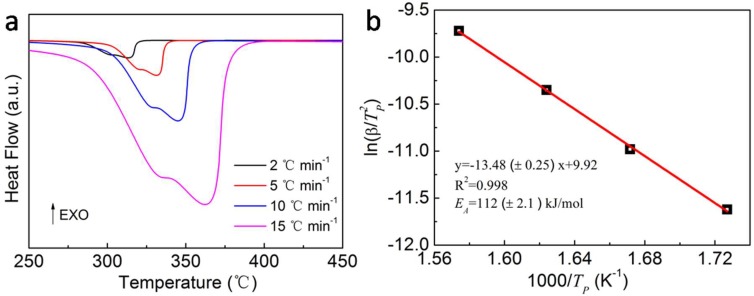
DSC plots of MgH_2_-4 wt.% Ni@C composites at heating rates of 2, 5, 10 and 15 °C min^−1^ (**a**), the Kissinger plots of MgH_2_-4 wt.% Ni@C composites (**b**).

The enhanced hydrogen desorption kinetics were then verified using the DSC measurements at various heating rates to calculate the activation energy of MgH_2_-4 wt.% Ni@C composites. The DSC plots of MgH_2_-4 wt.% Ni@C composites at heating rates of 2, 5, 10 and 15 °C min^−1^ are shown in Figure 5a. There is a broad peak at different heating rates corresponding to the decomposition of MgH_2_. The dehydrogenation peak temperatures, as recorded in Table 2, rise from 315 °C to 361 °C with the increase of the heating rate. For the MgH_2_ thermal decomposition reaction, the following equation can be used to calculate the activation energy [34]:

(1) $$\frac{d\left[\ln\left(\frac{\beta }{T_{P}^{2}}\right)\right]}{d\left(\frac{1}{T_{P}}\right)}=-\frac{E_{A}}{R}$$

where β is the heating rate, *T_P_* is the dehydrogenation peak temperature, *E*_A_ is the activation energy, R is the gas constant. In our work, the linear relationship between ln(β/*T_P_*^2^) and 1/*T_P_* has been presented, which is well-known as the Kissinger plot (Figure 5b). Hence, the *E*_A_ of the thermal decomposition for MgH_2_-4 wt.% composites is calculated approximately as 112 ± 2.1 kJ mol^−1^ using the value of the gas constant and the slope value (–13.48 ± 0.25) from the best linear fit of the Kissinger plot. The value of *E*_A_ is lower than the reported value of MgH_2_/-Ni_2_P/GNS (157 kJ mol^−1^) [35], MgH_2_-Ni_2_P (132.5 kJ mol^−1^) [36], MgH_2_-NiO (119.7 kJ mol^−1^) [36], and MgH_2_-MC10 (136 kJ mol^−1^) [37], which hints at the influence of the Ni@C materials on improving the hydrogen desorption kinetics of pure MgH_2_ materials.

## 3. Materials and Methods

Firstly, one-dimensional Ni@C nanorod materials were prepared following our initial work [38]. And commercial MgH_2_ powder (98 %) was bought from Alfa Aesar. Then the MgH_2_-*x* wt.% Ni@C composites (*x* = 0, 1, 2, 4 and 6) were manufactured through ball-milling. The specific ball-milling procedure was as follows: The big or small balls and the powders of MgH_2_ and Ni@C composites (with weight ratio of 40:1) were put into a steel jar. The steel jar was then fixed on the planetary ball mill and milled for 5 h at 450 rpm at the ambient temperature. The manipulations were conducted in a glovebox (O_2_ < 10 ppm; H_2_O < 10 ppm) to prevent moisture and oxygen.

The chemical constitution and fine structure of the MgH_2_-*x* wt.% Ni@C composites were carried out by X-ray diffraction (XRD, Rigaku D/Max-2500, Tokyo, Japan) and transmission electron microscopy (TEM, FEI Tecnai, Eindhoven, The Netherlands). The thermal decomposition properties of the composites were conducted by differential scanning calorimetry at 2, 5, 10 and 10 °C min^−1^ heating rates (DSC, Q20P, TA, Wilmington, DE, USA) and temperature-programmed desorption system with a 0.5 °C min^−1^ heating rate (TPD, PX200, Tianjin Golden Eagle Technology Co., Ltd., Tianjin, China). The high-purity Ar was used as a protective and sweeping gas during the DSC measurement. The Ar gas flow rate (30 mL min^−1^), the temperature range (50–450 °C) and the sample mass (7.5 ± 0.5 mg) were used for the DSC measurement. As for the TPD tests, the Ar gas flow rate was 35.1 mL min^−1^ and the temperature range was set at 50–500 °C. The sample mass was approximately 69 ± 2 mg in the TPD tests. The hydrogen absorption–desorption tests were characterized at different temperatures on a self-constructed Sievert’s device. 

## 4. Conclusions

In brief, the MgH_2_-*x* wt.% Ni@C (*x* = 0, 1, 2, 4 and 6) composites were prepared by ball-milling means. The hydrogen desorption properties of the MgH_2_-*x* wt.% Ni@C (*x* = 0, 1, 2, 4 and 6) composites were systematically investigated and the exact effects of the Ni@C materials addition on it have been discussed in this work. The experimental data suggest that the addition of the Ni@C materials can positively enhance the dehydrogenation kinetics of MgH_2_-Ni@C composites. Moreover, the optimized additive amount of the Ni@C materials was 4 wt.%, which is beneficial to decreasing the dehydrogenation temperature and maintaining an adequate hydrogen desorption amount. The MgH_2_-4 wt.% Ni@C composites can release 5.9 wt.% hydrogen in 5 min and 6.6 wt.% hydrogen in 20 min, whereas the pure MgH_2_ can only release 0.3 wt.% hydrogen in 20 min and 2.7 wt.% hydrogen in an even longer time of 120 min. The activation energy *E*_A_ of the MgH_2_-4 wt.% Ni@C composites was determined to be 112 kJ mol^−1^, which further demonstrates that the Ni@C could effectively enhance the hydrogen desorption kinetics of pure MgH_2_.

## Figures and Tables

**Table 1:** The dehydrogenation onset and peak temperatures of DSC plots for various MgH_2_-*x* wt.% Ni@C composites (*x* = 0, 1, 2, 4 and 6).

Sample	T_onset_ (°C)	T_peak_ (°C)
pure MgH_2_	343	358
MgH_2_-1 wt.% Ni@C	336	349
MgH_2_-2 wt.% Ni@C	314	325
MgH_2_-4 wt.% Ni@C	289	311
MgH_2_-6 wt.% Ni@C	286	307

**Table 2:** The dehydrogenation peak temperatures of DSC plots for MgH_2_-4 wt.% Ni@C composites at various heating rates.

Heating Rate (°C min^−1^)	Dehydrogenation Peak (°C)
2	315
5	331
10	345
15	361

## References

[1] Tollefson J. (2010). Fuel of the future. Nature.

[2] He T., Pachfule P., Wu H., Xu Q., Chen P. (2017). Hydrogen carrier. Nat. Rev. Mater..

[3] Schlapbach L., Zuttel A. (2001). Hydrogen-storage materials for mobile applications. Nature.

[4] Highfield J. (2015). Advances and recent trends in heterogeneous photo(electro)-catalysis for solar fuels and chemicals. Molecules.

[5] Yang J., Sudik A., Wolverton C. (2010). High capacity hydrogen storage materials: Attributes for automotive applications and techniques for materials discovery. Chem. Soc. Rev..

[6] Wang M., Chen L., Sun L. (2012). Recent progress in electrochemical hydrogen production with earth-abundant metal complexes as catalysts. Energy Environ. Sci..

[7] Nielsen T.K., Manickam K., Hirscher M. (2009). Confinement of MgH_2_ nanoclusters within nanoporous aerogel scaffold materials. ACS Nano.

[8] Aguey-Zinsou K.F., Ares-Fernandez J.R. (2010). Hydrogen in magnesium: New perspectives toward functional stores. Energy Environ. Sci..

[9] Stampfer J.F., Holley C.E., Suttle J.F. (1960). The Magnesium Hydrogen system. J. Am. Chem. Soc..

[10] Bardhan R., Ruminski A.M., Brand A. (2011). Magnesium nanocrystal-polymer composites: A new platform for designer hydrogen storage materials. Energy Environ. Sci..

[11] Wang Y., Li L., An C.H., Wang Y.J., Chen C.C., Jiao L.F., Yuan H.T. (2014). Facile synthesis of TiN decorated graphene and its enhanced catalytic effects on dehydrogenation performance of magnesium hydride. Nanoscale.

[12] Mukherjee D., Okuda J. (2018). Molecular magnesium hydrides. Angew. Chem. Int. Edit..

[13] Jeon K.J., Moon H.R., Ruminski A.M., Jiang B., Kisielowski C., Bardhan R. (2011). Air-stable magnesium nanocomposites provide rapid and high-capacity hydrogen storage without using heavy-metal catalysts. Nat. Mater..

[14] Hanada N., Ichikawa T., Fujii H. (2005). Catalytic effect of nanoparticle 3d-transition metals on hydrogen storage properties in magnesium hydride MgH_2_ prepared by mechanical milling. J. Phys. Chem. B.

[15] Pozzo M., Alfè D. (2009). Hydrogen dissociation and diffusion on transition metal (=Ti, Zr, V, Fe, Ru, Co, Rh, Ni, Pd, Cu, Ag)-doped Mg (0001) surfaces. Int. J. Hydrogen Energy.

[16] Jangir M., Jain A., Agarwal S., Zhang T., Kumar S., Selvaraj S., Ichikawa T., Jain I. (2018). The enhanced de/re-hydrogenation performance of MgH_2_ with TiH_2_ additive. Int. J. Energy Res..

[17] Bogdanović B. (1985). Catalytic synthesis of organolithium and organomagnesium compounds and of lithium and magnesium hydrides—applications inorganic synthesis and hydrogen storage. Angew. Chem. Int. Ed..

[18] Wang Z., Ren Z., Jian N., Gao M., Hu J., Du F., Pan H., Liu Y. (2018). Vanadium oxide nanoparticles supported on cubic carbon nanoboxes as highly active catalyst precursors for hydrogen storage in MgH_2_. J. Mater. Chem. A.

[19] Kumar S., Jain A., Miyaoka H., Ichikawa T., Kojima Y. (2017). Catalytic effect of bis(cyclopentadienyl) nickel (II) on the improvement of the hydrogenation dehydrogenation of Mg-MgH_2_ system. Int. J. Hydrogen Energy.

[20] Terzieva M., Khrussanova M., Peshev P. (1991). Dehydriding kinetics of mechanically alloyed mixtures of magnesium with some 3d transition metal oxides. Int. J. Hydrogen Energy.

[21] Hanada N., Ichikawa T., Fujii H. (2005). Catalytic effect of Ni nano-particle and Nb oxide on H-desorption properties in MgH_2_ prepared by ball milling. J. Alloy. Compd..

[22] Xie X., Chen M., Liu P., Shang J., Liu T. (2017). Highly hydrogen desorption properties of Mg-based nanocomposite at moderate temperatures: The effects of multiple catalysts in situ formed by adding nickel sulfides/graphene. J. Power Sources.

[23] Liang G., Huot J., Boily S., Neste A., Schulz R. (1999). Catalytic effect of transition metals on hydrogen sorption in nanocrystalline ball milled MgH_2_-Tm (Tm = Ti, V, Mn, Fe and Ni) systems. J. Alloys Compd..

[24] Milošević S., Kurko S., Pasquini L., Matović L., Vujasin R., Novaković N., Novaković J. (2016). Fast hydrogen sorption from MgH_2_-VO_2_(B) composite materials. J. Power Sources.

[25] Xia G., Tan Y., Chen X., Sun D., Guo Z., Liu H., Ouyang L., Zhu M., Yu X. (2015). Monodisperse magnesium hydride nanoparticles uniformly self-assembled on graphene. Adv. Mater..

[26] Barkhordarian G., Klassen T., Bormann R. (2006). Catalytic mechanism of transition-metal compounds on Mg hydrogen sorption reaction. J. Phys. Chem..

[27] Zhang J., Shi R., Zhu Y., Liu Y., Zhang Y., Li S., Li L. (2018). Remarkable synergistic catalysis of Ni-doped ultrafine TiO_2_ on hydrogen sorption kinetics of MgH_2_. ACS Appl. Mater. Interfaces.

[28] Zhang J., Li S., Zhu Y., Lin H., Liu Y., Zhang Y., Ma Z., Li L. (2017). Controllable fabrication of Ni-based catalysts and their enhancement on desorption properties of MgH_2_. J. Alloys Compd..

[29] An C., Liu G., Li L., Wang Y., Chen C., Wang Y., Jiao L., Yuan H. (2014). In situ synthesized one-dimensional porous Ni@C nanorods as catalysts for hydrogen storage properties of MgH_2_. Nanoscale.

[30] Huang X., Xiao X., Zhang W., Fan X., Zhang L., Cheng C., Li S., Ge H., Wang Q., Chen L. (2017). Transition metal (Co, Ni) nanoparticles wrapped with carbon and their superior catalytic activities for the reversible hydrogen storage of magnesium hydride. Phys. Chem. Chem. Phys..

[31] Chen G., Zhang Y., Chen J., Guo X., Zhu Y., Li L. (2018). Enhancing hydrogen storage performances of MgH_2_ by Ni nano-particles over mesoporous carbon CMK-3. Nanotechnology.

[32] Lillo-Ródenas M., Aguey-Zinsou K., Cazorla-Amoros D., Linares-Solano A., Guo Z. (2008). Effects of carbon-supported nickel catalysts on MgH_2_ decomposition. J. Phys. Chem. C.

[33] Jia Y., Yao X. (2017). Carbon scaffold modified by metal (Ni) or non-metal (N) to enhance hydrogen storage of MgH_2_ through nanoconfinement. Int. J. Hydrogen Energy.

[34] Kissinger H. (1957). Reaction kinetics in differential thermal analysis. Anal. Chem..

[35] Zhang Q., Xu Y., Wang Y., Zhang H., Wang Y., Jiao L., Yuan H. (2016). Enhanced hydrogen storage performance of MgH_2_-NiP/graphene nanosheets. Int. J. Hydrogen Energy.

[36] Zhang Q., Zang L., Huang Y., Gao P., Jiao L., Yuan H., Wang Y. (2017). Improved hydrogen storage properties of MgH_2_ with Ni-based compounds. Int. J. Hydrogen Energy.

[37] Liu G., Wang Y., Qiu F., Li L., Jiao L., Yuan H. (2012). Synthesis of porous Ni@rGO nanocomposite and its synergetic effect on hydrogen sorption properties of MgH_2_. J. Mater. Chem. A.

[38] An C., Wang Y., Jiao L., Yuan H. (2016). Mesoprous Ni@C hybrids for a high energy aqueous asymmetric supercapacitor device. J. Mater. Chem. A.

